# Prospective, crossover, comparative study of two methods of chlorhexidine bathing

**DOI:** 10.1017/ice.2024.243

**Published:** 2025-03

**Authors:** Richard Jordan Hankins, Luke Handke, Paul D. Fey, Ruth Jennifer Cavalieri, Kelly A. Cawcutt, Trevor Van Schooneveld, Elizabeth Lyden, Robin High, Mark E. Rupp

**Affiliations:** 1 Division of Infectious Diseases, University of Nebraska Medical Center, Omaha, NE, USA; 2 Pathology and Microbiology, University of Nebraska Medical Center, Omaha, NE, USA; 3 Epidemiology, University of Nebraska Medical Center, Omaha, NE, USA

## Abstract

**Background:**

Bathing intensive care unit (ICU) patients with chlorhexidine gluconate (CHG) decreases healthcare-associated infections (HAIs). The optimal method of CHG bathing remains undefined.

**Methods:**

Prospective crossover study comparing CHG daily bathing with 2% CHG-impregnated cloths versus 4% CHG solution. In phase 1, from January 2020 through March 2020, 1 ICU utilized 2% cloths, while the other ICU utilized 4% solution. After an interruption caused by the coronavirus disease 2019 pandemic, in phase 2, from July 2020 through September 2020, the unit CHG bathing assignments were reversed. Swabs were performed 3 times weekly from patients’ arms and legs to measure skin microbial colonization and CHG concentration. Other outcomes included HAIs, adverse reactions, and skin tolerability.

**Results:**

411 assessments occurred after baths with 2% cloth, and 425 assessments occurred after baths with 4% solution. Average microbial colonization was 691 (interquartile range 0, 30) colony-forming units per square centimeter (CFU/cm^2^) for patients bathed with 2% cloths, 1,627 (0, 265) CFUs/cm^2^ for 4% solution, and 8,519 (10, 1130) CFUs/cm^2^ for patients who did not have a CHG bath (*P* < .001). Average CHG skin concentration (parts per million) was 1300.4 (100, 2000) for 2% cloths, 307.2 (30, 200) for 4% solution, and 32.8 (0, 20) for patients without a recorded CHG bath. Both CHG bathing methods were well tolerated. Although underpowered, no difference in HAI was noted between groups.

**Conclusions:**

Either CHG bathing method resulted in a significant decrease in microbial skin colonization with a greater CHG concentration and fewer organisms associated with 2% CHG cloths.

## Introduction

Healthcare-associated infections (HAI) and multidrug-resistant organisms (MDRO) are a significant cause of morbidity and mortality in the hospital setting.^1^ Bacteria often colonize a patient’s skin and can become a source of infection.^2,3^ Chlorhexidine gluconate (CHG) is a broad-spectrum disinfectant that at low concentration causes osmotic disequilibrium and at high concentration enters into the bacterial cytoplasm to result in rapid cell death.^4,5^ The minimal effective concentration of CHG is 4.8–18.75 ppm (part per million).^6,7^ The effectiveness of CHG is amplified due to its residual activity, as it has been found to be effective for at least 6 hours following application.^8^ Bathing patients in intensive care units (ICU) with CHG has been shown to decrease colonization with MDROs, central-line associated bloodstream infection (CLABSI), and HAIs .^9–15^ Based on the reduction of CLABSI, CHG bathing is considered an essential practice in ICU patients >2 months of age.^16^ The best method for applying CHG to achieve adequate concentrations to decrease MDRO colonization and HAIs remains unclear. We planned our study to compare 2 commonly used CHG bathing regimens to better define optimal CHG bathing practices.

## Methods

### Setting and subjects

Two adult ICUs at a 718-bed academic medical center. The local Institutional Review Board deemed the project to be a quality improvement project, and informed consent was waived. The hospital quality improvement committee approved the study. The study included all patients 18 years of age or older, who were admitted into either the neurosciences ICU (NSICU) or surgical ICU (SICU) during the study period. The neuroscience ICU had both medical and surgical patients.

### Study design

**Figure 1.**
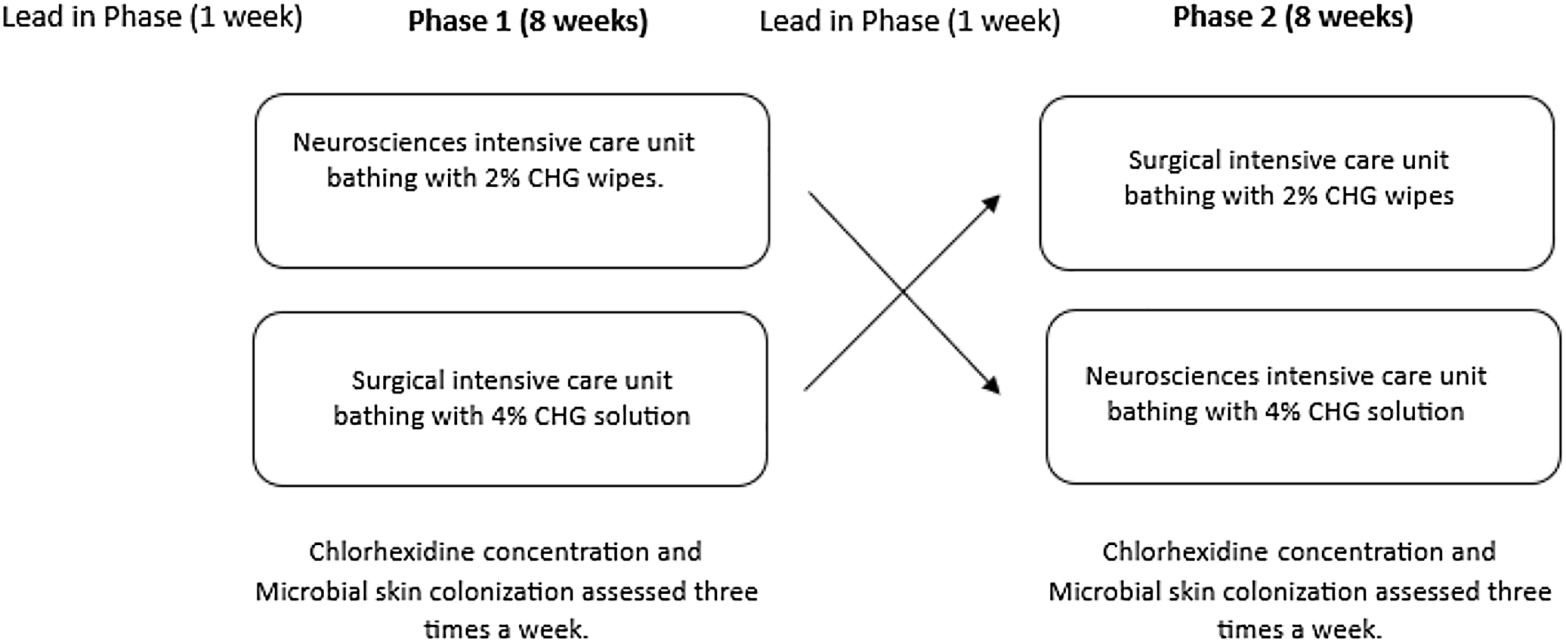
Schematic of the crossover study design.

Prospective crossover study comparing 2 methods of CHG patient bathing in 2 separate, although architecturally similar, ICUs. During phase 1, from January 2020 through March 2020, the nursing staff used the 4% solution in the NSICU, while the 2% cloths were used in the SICU. After phase 1 ended, and during the lead-in period to the second phase, all patient research and quality improvement projects with direct patient interaction were halted due to the coronavirus disease 2019 (COVID-19) pandemic. During phase 2, from July 2020 through September 2020, bathing regimens were reversed, and 2% cloths were used in the NSICU, and 4% solution was used in the SICU (Figure 1). There was a 1-week run-in period before each phase as the ICU staff became acclimated to the CHG bathing methodology.

Before each phase of the study, an educational program was conducted, and an informational slide set discussing the CHG bathing protocol was sent to all the nurses and medical assistants within the unit. ICU management required personnel responsible for patient bathing to review the educational program. Competency testing was not performed. Following the weeklong run-in period, samples were collected from the patient’s arms and legs 3 times per week for 12 weeks. The arm and leg sites were chosen for sampling despite other body sites (eg, inguinal) potentially having a higher microbial burden because it was felt to be less intrusive and thus more acceptable for patients and families for a quality improvement project with waived informed consent. On collection days, CHG and microbial colonization samples were collected from separate 5 cm × 5 cm templated areas on the arms and legs with premoistened cotton-tipped swabs, for a total of 4 swabs per patient. The swabs assessing microbial colonization were placed in sterile tubes containing 1 ml of sterile saline and promptly taken to a microbiology lab where they were vortex mixed, serially diluted with sterile saline, inoculated onto blood agar plates, incubated at 37**°**C for 48–72 hours, and assessed for microbial growth. A CHG-inactivating agent was not utilized on the microbial colonization swabs. A PhD-trained microbiologist (LH, PDF) assisted in the serial dilution, culturing, and colony counting assessments.

A colorimetric assay was used to determine the amount of CHG present on the skin.^17^ The colorimetric assay was performed by creating a standardized gradient of known CHG concentrations that were diluted in saline and applied to cotton-tipped swabs. The colorimetric assay gradient was then created by adding 100 µl of 1% cetyltrimethylammonium bromide to the swab, and then 20 µl of sodium bromide. 100 µl of 1% cetyltrimethylammonium bromide and 20 µl of sodium bromide were then applied to each CHG swab from patients and compared against the standardized colorimetric curve. A new standardized colorimetric curve was created each time the CHG swabs were assessed.^17,18^

To assess the patient’s skin condition and the potential impact of CHG bathing, the electronic medical record (EMR) was utilized to retrieve patient Braden scores assigned by nursing personnel. The Braden Scale is a standardized scoring system that was used daily by bedside nurses to assess dermatologic conditions. The Braden score ranges from 9 to 23 with a higher value indicating better skin condition and a lower risk for developing skin injury, specifically pressure injury.^19^ The EMR was also used to find the timing of the most recent CHG bath preceding the collection of CHG and microbial colonization skin swabs. Individuals who were identified through the EMR as not having a documented CHG bath were categorized separately as “no bath.” Antimicrobial use data was assessed post hoc from data routinely collected by the pharmacy and the antimicrobial stewardship program.

### Product

2% CHG-impregnated cloths (Sage) or a 4% CHG solution (Mölnlycke) was used to provide daily baths. Prior to the study, 4% CHG solution was the standard of care for daily patient baths in the NSICU and SICU (see Appendix 1 in supplement material).

### Study endpoints

The primary endpoints were microbial colonization at multiple anatomic locations and residual CHG levels on the skin. Secondary endpoints consisted of HAIs, skin conditions, and any adverse reactions associated with CHG bathing. Infection Control and Epidemiology Department personnel used Centers for Disease Control and Prevention National Healthcare Safety Network definitions and surveillance methods to define HAIs.^20^ HAIs that were monitored in the ICUs included CLABSI, catheter-associated urinary tract infection (CAUTI), *Clostridioides difficile* infection (CDI), methicillin-resistant *Staphylococcus aureus* bloodstream infection (MRSA BSI), ventilator-associated events (VAE) and vancomycin-resistant *Enterococcus* spp. bloodstream infection (VRE BSI).

### Statistical analysis

An a priori power analysis was conducted to determine differences that could be detected for both the residual chlorhexidine levels and the microbial burden. Group sample sizes of 160 patients bathed using 2% cloth and 160 patients bathed using the 4% solution achieve 90% power to detect a difference in mean remaining chlorhexidine of 373.0 ppm and a difference in mean bacterial burden of 932.5 CFU/cm^2^ assuming the within-group standard deviation was 1,000 ppm and 2,500 CFU/cm^2^, respectively, with a significance level of 0.05 using a two-sided Mann-Whitney test.^7,21^

For the data analyses, transformations of the arm, leg, and combined value of both limbs for both CHG assay (square root) and microbial (fourth root) were made to meet normality assumptions. With the transformed data, a linear mixed model (LMM) with random effects was employed to make comparisons of the means and compute *P*-values for combinations of pairwise differences. To account for multiple comparisons between group means, adjustments to the *P*-values were computed with simulation techniques.^22^ Results were presented as mean and interquartile ranges in the original units, but the *P*-values were based on the transformed data to meet the assumptions of the LMM model. Linear regression was performed to see if the time from the most recent bath to sample collection was predictive of CHG levels and microbial colonization. The mean Braden score was compared between the 3 groups using ANOVA. If the overall *P*-value was statistically significant, pairwise comparisons were adjusted using Tukey’s method. The association of HAI with the bathing method was assessed using Fisher’s exact test. All analyses were made using SAS/STAT software, version 15.2. *P* < .05 was considered statistically significant.

## Results

A total of 238 patients had CHG concentration and microbial colonization assessed in phase 1, and 231 patients were similarly assessed in phase 2. During phase 1, 132 patients were assessed in the NSICU (4% solution), and 106 patients were assessed in the SICU (2% cloths). During phase 2, samples were collected from 131 patients in the NSICU (2% cloths) and 100 patients in the SICU (4% solution). 411 assessments of CHG and microbial colonization were conducted after patients were bathed with 2% cloth, and 425 assessments of CHG and microbial colonization were conducted after patients were bathed with 4% solution (Table 1).

**Table 1.** Patient characteristics

	2% Chlorhexidine cloth	4% Chlorhexidine solution	No bath
	n=411 assessments	n=425 assessments	n=54 assessments
Different patients	204	211	53
Age, mean y (SD)	57.9 (16.6)	57.3 (18.1)	57.4 (19.2)
Sex, female, no (%)^a^	176 (42.9)	212 (49.9)	21 (39.6)
Race/ethnicity, no. (%)			
White	295 (71.8)	276 (64.9)	29 (53.7)
African American	58 (14.1)	54 (12.7)	4 (7.4)
Hispanic	45 (10.9)	84 (19.8)	6 (11.1)
Asian American	2 (0.5)	6 (1.4)	0 (0.0)
Other/unknown	11 (2.7)	5 (1.2)	15 (27.7)

a 1 patient was missing data for sex.

A total of 54 patients during the study had samples taken who had not yet received a CHG bath, had refused to have a bath, or had a contraindication (allergy). During phase 1, 30 individuals had samples taken without having a recorded CHG bath, while in phase 2, 24 individuals served as negative controls.

The overall average of microbial colonization values across both phases, assessed from both limbs, was 691 colony-forming units per square centimeter (CFU/cm^2^) for the 2% cloth, 1,627 CFU/cm^2^ for the 4% solution, and 8,519 CFU/cm^2^ for patients who did not have a CHG bath. There was a significant difference between the 2% cloth and 4% solution (*P* < .001), as well as each method compared to patients without a bath (*P* < .001). A significant difference was seen between 2% cloth and 4% solution for each limb and between each CHG method and no bath, except between 4% solution and patients without a bath when looking at only patients’ arms (*P* = .16) (Table 2).

**Table 2.** Outcomes from comparing 2% CHG cloth and 4% CHG solution

	2% CHG cloth	4% CHG solution	No bath
	n=411 assessments	n=425 assessments	n=54 assessments
Overall microbial colonization (IQR)	691 CFU/cm^2^ (0,30)	1,627 CFU/cm^2^ (0,265)^a^	8,519 CFU/cm^2^ (10,1130)^e^
Arm	559 CFU/cm^2^	1,236 CFU/cm^2^	2,336 CFU/cm^2^
Leg	824 CFU/cm^2^	2,018 CFU/cm^2^	14,588 CFU/cm^2^
Overall Chlorhexidine Concentration (IQR)	1,300.4 ppm (100,2000)	307.2 ppm (30, 200)^b^	32.8 ppm (0,20)^f^
Arm	1,058.7 ppm	274.2 ppm	18.3 ppm
Leg	1,543.3 ppm	340.2 ppm	47.2 ppm
Healthcare-associated infections^g^	7	6^c^	0
Adverse events	0	0	0
Braden score^g^	15.0	15.3^d^	18.4
Antimicrobial use (per 1,000 patient days)	773.0	718.4^h^	

Note. CHG, chlorhexidine gluconate; IQR, interquartile range; CFU, colony-forming unit.

a Comparison between CHG cloth and CHG solution *P* < .001.

b Comparison between CHG cloth and CHG solution *P* < .001.

c Comparison between CHG cloth and CHG solution *P* = .87.

d Comparison between CHG cloth and CHG solution *P* was not significant at < .05.

e Comparison between no bath and both CHG cloth and CHG solution *P* < .001.

f Comparison between no bath and both CHG cloth and CHG solution *P* < .001.

g n = 438 for 2% CHG cloth and n = 475 for 4% CHG solution.

h Comparison between CHG cloth and CHG solution antimicrobial use *P* = .93.

The overall means of the CHG dermal concentrations across both phase 1 and phase 2, including both limbs, were 1,300.4 ppm for the 2% cloth, 307.2 for the 4% solution, and 32.8 ppm for patients without a documented CHG bath. There was a significant difference between 2% cloth and 4% solution (*P* < .001), as well as each method and patients without a bath (*P* < .001). There was no significant difference in the amount of time between when patients had baths and when samples were collected between the 2 separate methods (*P* = .56).

During the study period, there were no reported adverse events or reactions to CHG bathing through a hospital incident reporting system. While monitoring for HAIs in the study, there were no CLABSIs, SSIs, or MRSA BSIs during the study period in either unit. There was 1 CAUTI, 6 VAE, and 6 CDI. There were 2 VAE, 1 CAUTI, and 3 CDI in patients treated with a 4% solution. There were 4 VAE and 3 CDI in patients treated with 2% cloth (Table 2). From July 2019–December 2020, 94.1% and 81.6% of patients received a daily CHG bath in the SICU and NSICU, respectively.

During phase 1, antimicrobial use (expressed as antimicrobial days per 1,000 patient days) was 671.9 in the NSICU and 1,038.8 in the SICU. During phase 2, the rate of antimicrobial use was 504.2 and 890.1 in the NSICU and SICU, respectively. Patients bathed with 2% cloth had antimicrobial use of 773.0, while patients with 4% solution had 718.4 (*P* = .93).

We also noted the cost of each method of bathing. Patients who were bathed with 2% cloth used 3 separate packs that contained 2 cloths each. The cost of each pack to our facility was $2.06. Patients who were bathed with 4% solution could receive 4 separate baths from the same 4-ounce bottle. The cost to our facility for 4-ounce bottles of 4% solution was $3.11. The direct cost per bath was $6.18 using 2% cloths, while the direct cost per bath with 4% solution was $0.78.

## Discussion

Daily CHG bathing has been convincingly shown to be an effective infection control measure to reduce MDRO transmission and HAIs in ICUs.^10^ Benefit has been shown from both CHG solution and CHG cloths, although the 2 methods have never been directly compared in a prospective, controlled trial. In our study, we compared 2% CHG cloths to 4% CHG solution with patients without a CHG bath serving as a negative control. We found that patients who received a CHG bath from either method had significantly fewer microbes on the skin when compared to patients who did not have a recorded bath and that the use of the 2% cloths was associated with a greater decrease in skin microbial colonization.

This reduction in microbial colonization correlated with a significantly higher concentration of CHG on the skin of patients who were bathed with 2% cloths compared to patients bathed with 4% solution. Notably, however, the average dermal CHG concentrations from both methodologies were significantly higher than the minimum effectiveness concentrations for CHG. Rhee *et al.* also found a correlation between higher CHG skin concentrations and less bacteria colonization.^23^ Previously evaluated minimum effectiveness concentrations ranged from 4.8 to 18.75 ppm, while the average concentration from the 2% cloth delivered 1,301 ppm and the 4% solution delivered 307.2 ppm. Based on this study, it appears that the higher CHG concentration provided by bathing with the 2% cloth results in a further reduction in microbial skin colonization compared to bathing with a 4% solution. It remains unclear however how high the optimal dermal CHG concentration should be to achieve maximal clinical benefit while minimizing potential adverse events such as dermal irritation or emergence of resistance.

In the protocol for performing the daily bathing, the nursing staff was instructed to follow manufacturer recommendations that were listed on each of the products. These instruct the personnel to rinse off the 4% solution after use, while not rinsing the skin of patients after they receive a bath with the 2% cloth. This rinsing step likely led to the significant difference that was seen in the CHG values between the 2 methodologies and possibly the difference that was seen in the residual microbial colonization. While the study was powered to assess the differences in the CHG concentration and microbial colonization, we also monitored the occurrence of HAIs. The study showed no significant difference between the 2 separate methodologies regarding HAIs, which was expected given the powering of the study. We also studied the possible dermatologic effect of both methods via bedside nurse-assessed Braden score. The study found that patients without a bath had a higher Braden score (lower risk for loss of skin integrity or development of pressure injury). This could be due to the individuals without a CHG bath simply being in the ICU for a shorter period and would thus be less prone to develop pressure injuries. Conversely, either CHG bathing method might be associated with a worsening Braden score. This should be examined more carefully in future studies on CHG bathing. There was no significant difference between the 4% solution and the 2% cloth regarding Braden scores. Reassuringly, there were no reports of adverse events associated with either CHG bathing method per the hospital adverse event reporting system.

The study had several strengths. To the best of our knowledge, this is the first study to directly compare the residual microbial colonization between the 2 most common methods of CHG patient bathing in a clinical setting. The crossover design allowed us to effectively evaluate the 2 separate bathing methodologies among 2 separate ICU populations. The study was also able to assess multiple sites for both CHG and microbial colonization, while also taking into consideration the timing of the patients’ last CHG bath.

The most notable limitation was the COVID-19 pandemic, which delayed the start of phase 2 of the study and led to changes in the patient care environment. Several infection control measures were instituted, including all staff wearing surgical masks and eye protection. Visitation to the units was also curtailed, although patients with COVID-19 were not housed in either of these units. Various measures to lower the inpatient census were instituted which may have affected nurse:patient ratios. This did not however affect the frequency of CHG daily bathing, although competency training was not assessed. Another limitation of the study was that the sampling only occurred for patients who were physically present. If patients were absent at the time of the ICU assessment, they were not sampled that day. A potential confounder of the microbial colonization and the HAIs was the antimicrobial use in each of the ICUs although there was no statistical difference in antimicrobial use rate seen between CHG methods. A CHG-inactivating agent was also not used on the microbial colonization swabs, so there is a possibility that the antimicrobial effect of CHG continued occurring after the swabs were taken. Staff documentation of bathing in the EMR was used to assess the conduct and timing of CHG patient bathing. We noted some of the patients who had not received a CHG bath per the record had detectable concentrations of CHG on their skin. This may indicate an error in patient charting or may indicate patient exposure to CHG off the study units. Also, CHG resistance was not assessed. Finally, surveillance cultures for MDROs were not performed nor were microbial cultures identified to species level and thus cannot be used to compare bathing methods.

In conclusion, we have seen that daily CHG bathing via either 2% cloths or 4% solution significantly reduced microbial colonization on the skin of ICU patients and was well tolerated. Although a formal cost-effectiveness study was not conducted, the acquisition cost associated with the 2% CHG cloths was substantially greater than the 4% CHG solution ($6.18 per bath for the 2% cloths vs $0.78 per bath for the 4% CHG solution). A large, cluster randomized trial to compare CHG bathing methods is justified to better define the effect of CHG bathing on MDRO acquisition and HAI occurrence, as well as cost-effectiveness and emergence of CHG resistance.

## Supporting information

Hankins et al. supplementary material Hankins et al. supplementary material

## Supplementary material

The supplementary material for this article can be found at https://doi.org/10.1017/ice.2024.243.
