# Supplementary material for: Prospective, crossover, comparative study of two methods of chlorhexidine bathing
Source: Infect Control Hosp Epidemiol. 2025 Jan 30;46(3):243–8. doi: 10.1017/ice.2024.243 (PMC11883645; doi:10.1017/ice.2024.243)
Supplement: Hankins et al. supplementary material [file S0899823X24002435sup001.docx]

**Supplemental Material**

**Appendix 1 Bathing Processes**

**2% Chlorhexidine Wipe**

Any visible debris on the patient's skin was removed prior to chlorhexidine bathing. The patients were cleaned of urine and stool prior to chlorhexidine bathing.

The nursing staff was instructed that a bath with the 2% chlorhexidine wipes would require 6 total wipes, that were packaged in packets of two. The nursing staff placed three packages in the Sage Warmer Model 7939 about one hour prior to a patient’s bath. The wipes were then removed from the warmer. One packet was opened, and the first wipe (#1) was used on the neck, chest, abdomen, and groin. This wipe was then discarded. The next wipe was removed from the packet and cleaned the back and buttocks. This wipe was discarded, and the second packet then opened. The next wipe (#3) then cleaned the left arm moving distally. The next wipe (#4) then cleaned the right arm. This was then discarded, and the third packet opened. The next wipe (#5) then cleaned the left leg moving distally. This was discarded, and the final wipe was then used to clean the right leg. Wipes were not used on open wounds. No rinsing of the chlorhexidine occurred after bathing.

If a chlorhexidine packet was left in the warmer for 72 hours, it was discarded.

**4% Chlorhexidine Solution.**

Any visible debris on the patient's skin was removed prior to chlorhexidine bathing. The patients were cleaned of urine and stool prior to chlorhexidine bathing.

The nursing staff placed 8 dry washcloths into a plastic bag. The bag was filled with just enough warm tap water to moisten the washcloths. They were instructed the washcloths would not be dripping. The plastic bag with moistened washcloths was to remain soap-free throughout the bath. The soap was to be applied to the washcloth once it was removed from the plastic bag.

The first clean washcloth (#1) from the plastic bag and cleaned the region around the patient’s eyes/periorbital tissues with plain warm water without the CHG cleansing solution. The next clean washcloth (#2) from the bag had 2 pumps of the CHG solution applied. One arm was cleaned from distal to proximal areas (fingers to axilla). This was repeated by applying 2 pumps of the CHG foam soap using the same washcloth for the other arm to wash. The washcloth (#2) was then discarded in a laundry bag. The next clean washcloth (#3) from the bag had 2 pumps of the CHG solution. This cleaned the chest, including skin folds under the patient’s breast, and abdomen, including umbilicus and abdominal folds as indicated. This was then discarded in a laundry bag. The next clean washcloth (#4) from the bag had 2 pumps of the CHG solution applied. This cleaned ankle to knee and from knee to thigh, and then the foot including between the toes. The next washcloth (#5) repeated the process of applying 2 pumps of the CHG solution to the washcloth for the other leg and foot.

The CHG solution was then rinsed off of the arms, abdomen, and legs with a washcloth (#6). The next clean washcloth (#7) had 2 pumps of the CHG solution applied, and cleaned back from neck to buttocks. The last washcloth (#8) then rinsed the same area.
